# Population risk factors for COVID-19 deaths in Nigeria at sub-national level

**DOI:** 10.11604/pamj.supp.2020.35.131.25258

**Published:** 2020-08-04

**Authors:** Zubaida Hassan, Muhammad Jawad Hashim, Gulfaraz Khan

**Affiliations:** 1 Department of Microbiology, School of Life Sciences, Modibbo Adama University of Technology, Yola, Nigeria,; 2 Department of Medical Microbiology and Immunology, College of Medicine and Health Sciences, United Arab Emirates University, United Arab Emirates,; 3 Department of Family Medicine, College of Medicine and Health Sciences, United Arab Emirates University, United Arab Emirates

**Keywords:** SARS-CoV-2, COVID-19, mortality, epidemiology, risk factors, Nigeria, Africa

## Abstract

**Introduction:**

Nigeria is the most populous country in the African continent. The aim of this study was to analyze risk factors for COVID-19 prevalence and deaths in all 6 geopolitical regions and 37 States in Nigeria.

**Methods:**

we analyzed the data retrieved from various sources, including Nigeria CDC, Nigeria National Bureau of Statistics, Unicef-Nigeria multiple indicator cluster survey and the Institute of Health Metrics and Evaluation, University of Washington. We examined 4 clinical risk factors (prevalence of TB, HIV, smoking and BCG vaccination coverage) and 5 sociodemographic factors (age ≥65, population density, literacy rate, unemployment and GDP per capita). Multivariate modeling was conducted using generalized linear model.

**Results:**

our analysis showed that the incidence of confirmed COVID-19 cases differed widely across the 37 States, from 0.09 per 100,000 in Kogi to 83.7 in Lagos. However, more than 70% of confirmed cases were concentrated in just 7 States: Lagos, Abuja, Oyo, Kano, Edo, Rivers and Delta. Case mortality rate (CMR) also varied considerably, with Lagos, Abuja and Edo having CMR above 9 per million population. On bivariate analysis, higher CMR correlated positively with GDP (r=0.53) and to a lesser extent with TB (r=0.36) and population density (r=0.38). On multivariate analysis, which is more definitive, States with higher HIV prevalence and BCG coverage had lower CMR, while high GDP States had a greater CMR.

**Conclusion:**

this study indicates that COVID-19 has disproportionately affected certain States in Nigeria. Population susceptibility factors include higher economic development but not literacy or unemployment. Death rates were mildly lower in States with higher HIV prevalence and BCG vaccination coverage.

## Introduction

Prior to the emergence of the current COVID-19 pandemic, the world had witnessed the outbreak of two other β-coronaviruses; SARS-CoV-1 (severe acute respiratory syndrome coronavirus) in 2003 [1] and MERS-CoV (Middle East respiratory syndrome coronavirus) in 2012 [2]. Coronaviruses are a large group of viruses known to cause respiratory illnesses in humans with symptoms ranging from mild to severe diseases [3]. In December 2019 a new coronavirus outbreak was reported in China [4]. The new virus, named as SARS-CoV-2, spread rapidly from country to country causing unprecedented impact on health, economy, and quality of life of communities. Surprisingly, economically rich countries such as Italy, the US and UK, in spite of their well-established healthcare systems, experienced the highest burden of infection and deaths. Numerous factors, including, delayed public health responses, limited testing, inadequate contact tracing and quarantine measures, as well population demographics and disease comorbidities are believed to have contributed to the high burden in these countries [5,6].

The African continent reported its first case on 14^th^ February 2020 in Egypt [7]. Nigeria recorded her first COVID-19 case on 27^th^ February, becoming the second country in Africa with the disease [7]. The index case was a 44-year old Italian citizen who flew into Lagos International Airport from Milan on 25^th^ February. Indeed, Nigeria´s first dozen cases of COVID-19 were all linked to travel history from endemic countries. Nigeria´s response included suspension of flights, supervised self-isolation for returnees, continuous contact tracing and restrictions on interstate travels. Other measures included nationwide closure of all tertiary, secondary and primary schools, prohibition of religious and other high-density gatherings and social distancing [8]. As of June 30, 2020, Nigeria recorded 25,694 COVID-19 cases, 590 deaths and 9,746 recoveries. However, the distribution and burden of morality from COVID-19 varied significantly from region to region and indeed from State to State. The aim of this study was to analyze at the State and regional levels, the population risk factors that could explain these variations in COVID-19 burden in Nigeria.

## Methods

**Data sources:** data for prevalence and case fatality (CFR) of COVID-19 was collected for all 6 geopolitical regions and all 37 States in Nigeria from the beginning of the outbreak until 30^th^ June 2020. COVID-19 data was obtained from the Nigeria Centre for Diseases Control (NCDC) [9]. Prevalence and CFR of COVID-19 cases were analyzed in the context of different clinical and socio-demographic risk factors. Data on State´s total population, unemployment, literacy, GDP per capita and cases of TB was obtained from the Nigeria National Bureau of Statistics (NNBS) [10]. We calculated the socio-demographic parameters, namely, population density, elderly population (65 years and above), and case mortality rates (CMR), from the raw data obtained from the NCDC and NNBS. BCG vaccination coverage (the oldest available data from 2000 was used, as the risk of COVID-19 mortality is highest in older cohorts), and HIV prevalence were obtained from the Local Burden of Disease (LBD) dataset from Institute of Health Metrics and Evaluation, University of Washington, Seattle [11]. State-wise data on smoking prevalence was obtained from Unicef-Nigeria multiple indicator cluster survey (MICS) [12].

**Statistical analysis:** statistical data analysis was conducted using Jamovi version 1.1.7. Bivariate analysis included correlation analysis of risk factors with CMR in the 37 States. One-way ANOVA was used to compare the risk factors in 7 higher burden States versus all other States. Mean differences in these two groups were compared using post hoc Tukey´s adjusted p values. Lastly, multivariate analysis was carried out using generalized linear model (GAMLj module) based on Poisson distribution with the log link function for CMR (the dependent variable). Loglikelihood ratio tests were used to evaluate the predictive performance of each covariate while R-squared was maximized for overall model error reduction. No States were excluded. Missing data were minimal for COVID-19 metrics.

## Results

**Figure 1:**
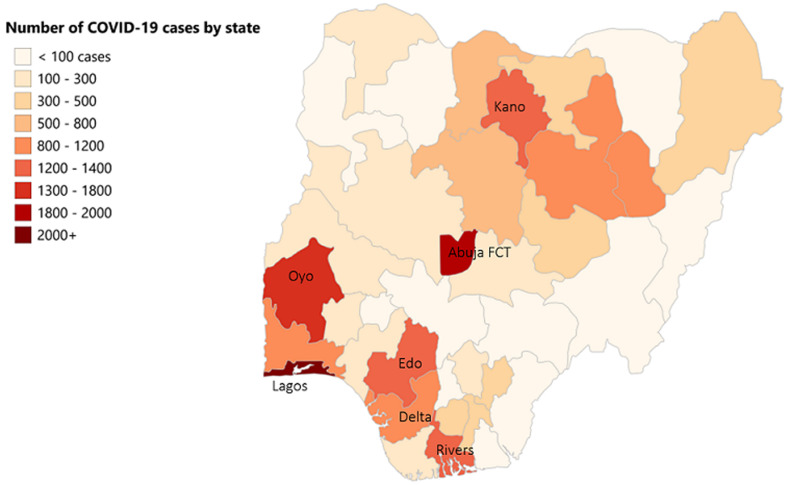
distribution of COVID-19 confirmed cases across the 37 States in Nigeria (as of 30^th^ June 2020)

**Descriptive analyses:** we analyzed the data for all 6 geopolitical zones and all 37 States of Nigeria including the federal capital territory (FCT) (Annex 1). The total number of laboratory-confirmed cases increased rapidly from 139 persons in March 2020, to 1,932 in April, escalating to 10,162 cases by May 2020 and 25,694 by the end of June. COVID-19 mortality rates rose from an average of 0.05 deaths per State in March 2020 to 1.56 deaths in April, going up to 7.76 deaths in May 2020 and 16.0 in June. Case fatality rates per 100 confirmed cases (by 30^th^ June 2020) ranged from less than one to a maximum of 13.6 in Yobe State. The greatest burden of deaths were seen in South-South, North-West and South-West geopolitical zones, reporting 117, 120 and 185 lives lost, respectively. These regions also had the highest number of confirmed cases at 3769, 3163 and 13178, respectively (Figure 1). From the 37 States, Lagos (located in South-West zone) had the largest number of confirmed cases and deaths (10,510 and 128, respectively, by 30^th^ June 2020) (Figure 1). Comparison of the 7 the States with the highest COVID-19 confirmed cases, namely Lagos, Abuja FCT, Oyo, Kano, Edo, Rivers, and Delta with all other States revealed that the high burden States had significantly higher number of total deaths. This remained significant when mortality was calculated by per million population, CMR (Table 1).

**Table 1:** comparison of Nigerian States with high COVID-19 burden with other States (as of 30^th^ June 2020)

	7 High COVID-19 burden States with CMR ≥ 5 (Lagos, Abuja FCT, Oyo, Kano, Edo, Rivers, Delta)	Other 30 States	Difference	p-value^*^
**Totals of COVID-19 metrics**				
Total confirmed cases	15,742	9,952	1,905	0.007
Total deaths	303	287	33	< 0.001
**Means of COVID-19 metrics**				
CFR per 100 confirmed cases	3.89	4.08	-0.19	0.888
CMR per million population	7.33	1.76	5.57	< 0.001
**Clinical risk factors**				
BCG coverage (%, 2000)	56	53	3	0.763
Tuberculosis prevalence	3,429	1,941	1,488	0.618
HIV prevalence	3.7	2.9	0.8	0.300
Cigarette smoking	12.3	12.8	-0.5	0.882
**Demographic factors**				
Elderly 65+ years	2.06	2.47	-0.41	0.233
Population density	760	357	403	0.407
Literacy rate (%, 2016)	71.5	60.3	11.2	0.419
Unemployment (%, 2018)	27.4	22.3	5.0	0.123
GDP per capita (USD, 2007)	2,849	1,769	1,079	0.076

* P values for post-hoc Tukey tests based on one-way ANOVA.

**Figure 2:**
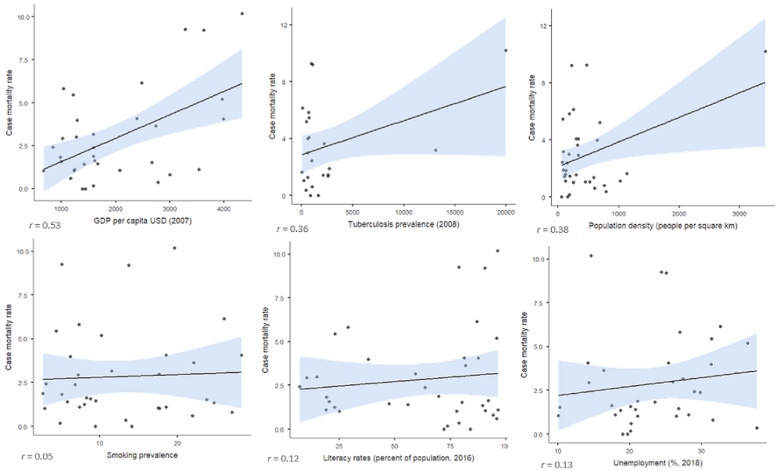
associations of COVID-19 case mortality rates with selected clinical and demographic variables in Nigerian States (as of 30^th^ June 2020)

**Bivariate correlations:** higher CMR per million population correlated with GDP per capita (r = 0.53), and to a lesser extent with population density (r = 0.38) and TB prevalence (r = 0.36), but not with smoking, literacy or unemployment (r < 0.15) (Figure 2). As bivariate correlations can be spurious due to hidden confounding variables, a multivariate analysis was carried out.

**Figure 3:**
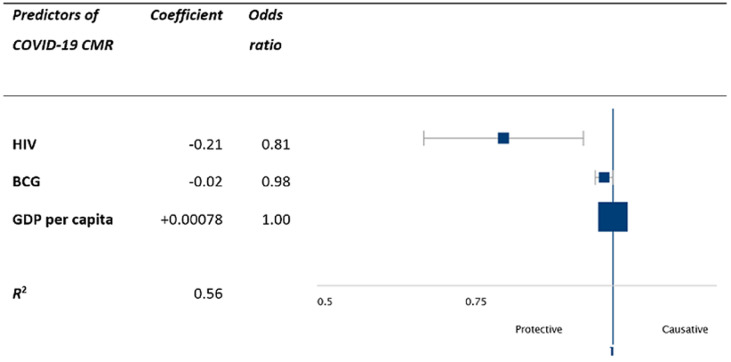
multivariate analysis of predictors of COVID-19 case mortality rates in Nigerian States (as of 30^th^ June 2020)

**Multivariate analysis:** multivariate modelling was used to analyze the relative contribution of predictor variables for CMR in Nigerian States. Variation of CMR across the 37 States was predicted by 3 covariates (p < 0.05 for all variables; adjusted R^2^, 0.56). These variables were prevalence of HIV (adjusted odds ratio (OR), 0.81, 95% CI, 0.68-0.95), GDP per capita (OR 1.0) and BCG vaccination (OR 0.98, CI 0.97-1.0). HIV prevalence had a protective effect, whilst GDP and BCG vaccination were also statistically significant but their protective effect was small (Figure 3). Modelling for CFR as the outcome variable did not uncover any variable that was a statistically significant predictor.

## Discussion

The current COVID-19 pandemic has claimed the lives of hundreds of thousands of individuals worldwide, but at a varying degree. Different countries have taken different policy decisions based on their specific capabilities and needs in responding to the pandemic. These actions have clearly had different impact on the rate of infection and death associated with COVID-19. Since the death toll from this pandemic continues to grow, there is an urgent need to identify risk factors that predispose to severe disease outcomes. In low-income countries with nascent healthcare systems and limited resources, knowing who is at increased risk of severe disease is even more compelling [7,13,14]. In this study, we have analyzed the population risk factors of COVID-19 in Nigeria at the subnational State level.

Previous studies have suggested that BCG vaccination may have a protective role in COVID-19 disease severity [15-17]. Although BCG was developed to protect against *Mycobacterium tuberculosis*, the causative agent of tuberculosis, it has been reported to induce cross-protective trained immunity against a number of viral and bacterial pathogens [18-20]. In this study, we found that low BCG vaccine coverage correlated with higher COVID-19 mortality. This was further supported by the observation that high TB prevalence correlated with higher COVID-19 mortality. However, these associations were weak. For BCG vaccination, we used data from the year 2000. Since most of the confirmed cases of COVID-19 in Nigeria have been reported in the age group 21-50 years [9], using data for BCG vaccination coverage from 1970 to 2000 would have provided a more valid analysis and perhaps a stronger correlation. Unfortunately, State level data prior to 2000 was not available. Despite no direct evidence from randomized case-controlled studies, epidemiological analysis from several countries indicate that the COVID-19 prevalence, progression and mortality are negatively correlated with BCG coverage [15,21,22]. Higher deaths were observed in countries that have stopped BCG vaccination [15]. However, a number of pertinent questions regarding the potential protective role of BCG for COVID-19 severity remain unknown. For example, is the BCG protective effect age and gender dependent? What is the protective duration of BCG vaccination? Is the protective effect dependent on the strain of BCG vaccine?

Another interesting observation from this study was the negative correlation between HIV prevalence and COVID-19 mortality. Although it was expected, and with good reason, that HIV patients may succumb to more severe COVID-19 infection and mortality, our data does not appear to support this. Indeed, a few recent reports also indicate that HIV patients are at no greater risk of severe COVID-19 compared to the general population [18-20,23]. Why people with HIV do not develop more severe COVID-19 remains puzzling. One suggestion is that some of the antiretroviral drugs used for the treatment of HIV could have protective effect against SARS-CoV-2. However, this remains controversial and unproven. Another possibility is that people with HIV are more cautious about social distancing, and hence are less likely to get infected with SARS-CoV-2 in the first place.

A number of studies have reported that old age is a risk factor for COVID-19 severity [24-26]. Surprisingly, our analysis did not show this correlation. The age group most affected by COVID-19 in Nigeria, as of June 30^th^ 2020, were 21-50 years with a peak infection among 31-40 years [9]. This is in contrast to reports from China where 87% of confirmed cases were aged between 30 and 79 years old [27] with a median age of 48 to 58 years [28]. A similar pattern was observed in Italy [29] and the US [27]. One possible explanation for the difference could be due to the specific demographics of Nigeria. Those aged 65 and above constituted only 2% of the population as compared to 22.5% in Italy, for example. As for gender, men were most affected with COVID-19, consistent with reports from other countries [24-26,30]. One speculation is that men smoke more than women in Nigeria. Smoking is a risk factor for developing more severe forms of COVID-19 [27] probably because it affects the lungs rendering it more susceptible to the infection. However, in our multivariate analysis, smoking prevalence did not correlate with COVID-19 deaths, at least in Nigerian State level analysis. We have found that factors like literacy, unemployment or smoking were not predictive of either incidence or fatality of COVID-19. It possible that in Nigeria, other risk factors may have had a more significant impact on COVID-19 incidence and fatality. These may include observing recommended COVID-19 precautionary measures such as social distancing, regular handwashing with soap and water, avoiding SARS-CoV-2 infected or suspected person and use of face masks where necessary [8,31]. It is also possible that poorer States with low literacy and high unemployment did fewer tests and had fewer foreign visitors with COVID-19.

## Conclusion

It is apparent that SARS-CoV-2 infects both rich and literate individuals and countries globally. Nonetheless from our analyses, States' GDP per capita and population density appeared to be moderate risk factors for COVID-19 CMR per million population whereas BCG coverage and HIV prevalence appeared to afford some protection. This study serves as a scaffold for understanding population risk factors for COVID-19 deaths in Nigeria. Policymakers and healthcare professionals can benefit from evidence-based recommendations to guide their decisions in the evolving pandemic.

### What is known about this topic

- COVID-19 pandemics has affected all countries worldwide;
- Countries in the African continent with poorly developed healthcare systems were predicted to be particularly vulnerable to COVID-19 pandemic, but to-date, this has not happened;
- There is considerable evidence that COVID-19 severity and death is related various clinical and socio-demographic risk factors.

### What this study adds

- This is the first study of its kind to examine the association of clinical (prevalence of TB, HIV, smoking and BCG vaccination coverage) and socio-demographical risk factors (age ≥65, population density, literacy rate, unemployment and GDP per capita) for COVID-19 deaths in Nigeria at regional and State level;
- Our findings indicate that COVID-19 has disproportionately affected certain States in Nigeria, in particular those States like Lagos and Abuja which are the most affluent;
- Interestingly, States with higher HIV prevalence and BCG coverage had lower CMR. Case control studies are required to assess the effectiveness of BCG vaccination in protecting against COVID-19.

## Annex

Annex 1: raw data (PDF-378Ko)
